# Assessment of Hemostasis after Plasma Exchange Using Rotational Thrombelastometry (ROTEM)

**DOI:** 10.1371/journal.pone.0130402

**Published:** 2015-06-29

**Authors:** Gerold Thölking, Rolf Mesters, Ralf Dittrich, Hermann Pavenstädt, Philipp Kümpers, Stefan Reuter

**Affiliations:** 1 Department of Medicine D, Division of General Internal Medicine, Nephrology and Rheumatology, University Hospital of Münster, Albert-Schweitzer-Campus 1, Münster, 48149, Germany; 2 Department of Medicine A, Division of Hematology and Oncology, University Hospital of Münster, Albert-Schweitzer-Campus 1, Münster, 48149, Germany; 3 Department of Neurology, University Hospital of Münster, Albert-Schweitzer-Campus 1, Münster, 48149, Germany; University of Toledo, UNITED STATES

## Abstract

**Background:**

Therapeutic plasma exchange (TPE)-based protocols immediately before cadaveric donor kidney transplantation have been extensively used in highly sensitized recipients. Plasma is generally preferred over human albumin as replacement fluid to avoid depletion of coagulation factors and perioperative bleeding. The aim of this study was to estimate bleeding risk after TPE replaced with albumin using rotational thromboelastography (ROTEM).

**Methodology:**

Ten patients without overt coagulation abnormalities underwent TPE. Standard laboratory coagulation tests (thromboplastin time, activated partial thromboplastin time (aPTT), international normalized ratio (INR), thrombin clotting time, fibrinogen levels and antithrombin activity) were compared with thrombelastometry analysis (EXTEM and INTEM tests) before and after TPE.

**Principal Findings:**

TPE significantly reduced fibrinogen levels (482 ± 182 vs. 223 ± 122 mg/dL), antithrombin activity (103 ± 11 vs. 54 ± 11 %), and prolonged aPTT (28 ± 3 vs. 45 ± 8 s), thromboplastin time (108 ± 11 vs. 68 ± 11 %), INR (0.95 ± 0.06 vs. 1.25 ± 0.16), and thrombin clotting time (18 ± 2 vs. 20 ± 3 s). INTEM and EXTEM analyses revealed significantly prolonged clot-formation time and reduced maximum clot firmness.

**Conclusions/Significance:**

TPE replaced with albumin induces significant changes in global hemostasis parameters thus potentially increasing bleeding risk. Therefore, pretransplant TPE should be considered carefully in indicated patients before kidney transplantation. The role of the ROTEM point-of-care test to estimate the risk of bleeding in renal transplantation needs to be evaluated in further studies.

## Introduction

Therapeutic plasma exchange (TPE) is used as a rescue therapy for antibody-mediated diseases but causes far more effects than simple antibody depletion [1, 2]. Mainly in the USA and Japan, TPE is a standard procedure in desensitization of patients before ABO incompatible (ABOi) renal transplantation (RTx) [3, 4]. It is also employed for depletion of antibodies in high-risk sensitized patients immediately before RTx [5]. In general, two different kinds of replacement solutions exist [6]. In most cases, use of human serum albumin solution (5%) in saline is preferred due to better safety and side-effect profile although this leads to a depletion of most plasma proteins, especially when repeatedly applied. Albumin preparations are processed for convenient use (easy handling and storage, decreased need for IV calcium), virus inactivation and are well tolerated by most patients [7–9]. Plasma handling is more complex (blood type specific ordering, thawing, greater frequency of hypocalcemia due to citrate anticoagulant in the plasma) and is more likely to cause allergic reactions. However, because plasma replaces all plasma proteins it is potentially the solution of choice in the perioperative management of the kidney transplant recipient to avoid removal of clotting factors and subsequent increased bleeding risk [10].

Besides its effects on clotting factors, cellular blood components i.e. platelets are also influenced by TPE [11, 12]. In the case of RTx patients, TPE causes changes in an already profoundly changed coagulation system of patients with renal failure [13]. Thus, perioperative bleeding management and monitoring of coagulation activity of RTx recipients is challenging and evaluation of standard test like activated Partial Thromboplastin Time (aPTT), Prothrombin Time (PT, Quick)/International Normalized Ratio (INR) might be inferior to point-of-care (POC) assays that evaluate the viscoelastic properties of blood i.e. including thrombelastography and rotational thromboelastometry (ROTEM) [14]. These tests can assess the whole clotting process at the bedside. Coagulation is tested in whole blood, and includes interactions with platelets and red cells, thereby providing additional information on patient`s coagulation status to estimate patient`s bleeding risk more precisely [15]. In fact, if ROTEM analysis is unremarkable, one should seek for surgical bleedings.

As aforementioned, the usage of albumin for TPE in sensitized patients receiving cadaveric RTx immediately before surgery would have several advantages compared to plasma use. However, due to possible changes on global hemostasis, it`s effects need to be evaluated in this regard. Thus, we herein evaluate the effect of TPE with albumin on coagulation by standard coagulation tests and ROTEM analysis.

## Material and Methods

### Study population

From 2011 to 2013 ten patients underwent TPE treatment due to neurologic indications in our center. Clinical data were collected prior to first TPE. Data of all patients were anonymized and de-identified prior to analysis. Our study was performed in accordance with the declaration of Helsinki and approved by the local ethics committee (Ethik Kommission der Ärtzekammer Westfalen-Lippe und der Medizinischen Fakultät der Westfälischen Wilhelms-Universität, No. 2011-601-f-S). Enrollment was performed after written informed consent was given by all participants prior to first TPE for recording their clinical data and use in anonymized analysis.

### Therapeutic plasma exchange (TPE)

According to the standard protocol of our center, prior to first TPE, a central venous dialysis catheter was inserted into the internal jugular vein. TPE was performed using a COM.TEC cell separator (Fresenius Hemo-Care GmbH, Bad Homburg, Germany). All patients were treated five times with 2L albumin 5% (0.4 to 1.0 plasma volumes) per session using an interval of 1–2 days. In contrast to regimes of other groups, we did not use (additional) fresh frozen plasma in our patients. Blood flow rates were 50–70 mL per min. All patients underwent regional pre-centrifugal anticoagulation with citrate (ACD-A, Hemonetics, Hemonetics corporation, Braintree, Massachusetts, USA, 1:20 ml/min in relation to blood flow) followed by post-centrifugal calcium (Calcium gluconate 10%, B. Braun Melsungen AG, Melsungen, Germany) application (started with 15 mL per h and adaption to clinical symptoms of the patients).

### Laboratory tests

Ethylenediaminetetraacetic acid (EDTA) and citrated blood samples were obtained immediately before and after the first procedure from the central venous catheter. Blood count was carried out from EDTA blood and included red blood cells, hemoglobin (Hb), hematocrit, white blood count (WBC) and platelets. Conventional coagulation parameters comprised fibrinogen, aPTT, INR, antithrombin (AT) and thrombin time (TT).

### ROTEM thrombelastographic analysis

All samples were analyzed within 90 min after collection using a ROTEM Coagulation Analyzer according to the manufacturer’s protocol (Pentapharm, Munich, Germany). EXTEM (tissue factor activation) and INTEM (ellagic acid/phospholipid activation) were performed.

**Fig 1:**
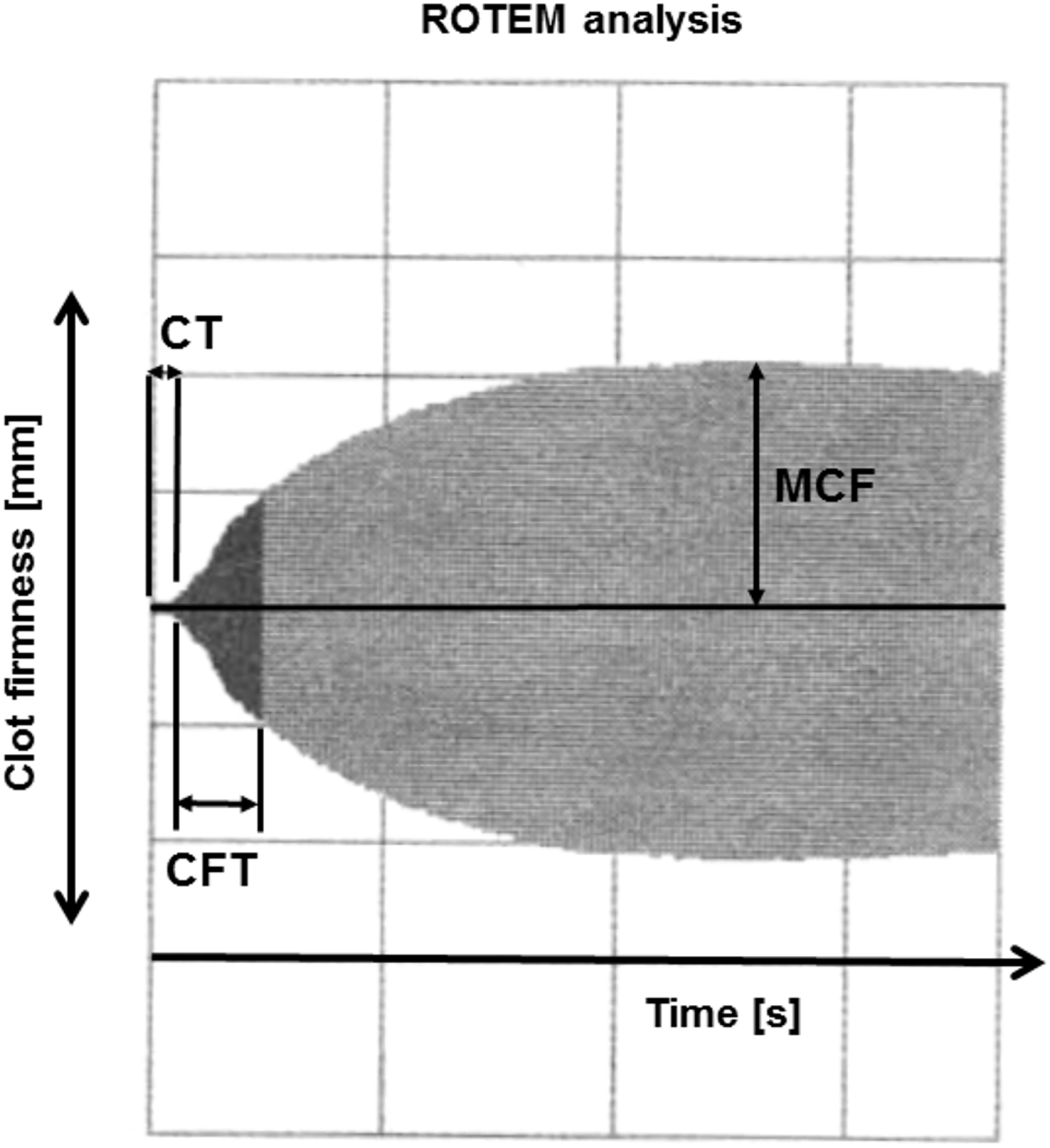
Thrombelastogram obtained by rotational thromboelastography (ROTEM). ROTEM analysis is a point-of-care assay that allows the assessment of the complete clotting process. Functional coagulation parameters like clot-formation time (CFT), maximum clot firmness (MCF), and clotting time (CT) are tested in whole blood.

The following ROTEM parameters were analyzed: clot-formation time (CFT), maximum clot firmness (MCF), and clotting time (CT). CT is the latency time from adding the reagent to blood until initiation of clotting. CT is influenced by activities of conventional coagulation factors. CFT is the time from initiation of clotting until a clot firmness of 20 mm has been reached. CFT is also influenced by activities of coagulation factors but also includes platelet count/function, thrombin formation, fibrin precipitation, fibrinogen and hematocrit. MCF represents absolute strength of the fibrin and platelet clot. MCF is affected by fibrin and fibrinogen concentration, platelet count/function, thrombin concentration, factor XIII, and hematocrit. Fig 1 illustrates the parameters in a thrombelastogram obtained by ROTEM.

### Statistical analysis

Statistical analyses of data and figure preparation were performed using GraphPad Prism 4.0 (GraphPad Software, La Jolla, CA, USA).

The Wilcoxon signed rank test was used for all blood values and coagulation parameters. All tests were two-sided and P-values were considered significant if <0.05.

## Results

### Patient characteristics

Descriptive statistics of the patients who underwent TPE with albumin are presented in Table 1. The patient cohort consisted of 6 male and 4 female Caucasian patients with a median age of 52.4 (range 21.7–62.7) years. 0.4 to 1.0 plasma volumes were processed during each session. Mean exchanged plasma volume was 27.0±5.2 mL/kg BW (range 16.4–33.9 mL/kg BW). No serious adverse events due to TPE or during the entire hospital stay were observed.

**Table 1:** Patient characteristics.

Age (yr)	52.4 (21.7–62.7)
Gender (m/f)	6/4
Weight (kg)	72 (55–122)
Height (m)	1.73 ± 0.12
BMI (kg/m^2^)	24 (20–38)
Neurological disorder	
Multiple sclerosis	3
Guillain-Barré syndrome	2
Limbic encephalitis	2
Neuromyotonia	1
Myasthenia gravis	2

### Laboratory parameters before first TPE

The conventional coagulation parameters, the blood count and ROTEM analysis are shown in Table 2. Prior to TPE all assessed parameters were within the normal range except fibrinogen, which showed an increased mean value of 482±182 mg/dL.

**Table 2:** Coagulation parameters, ROTEM analysis and blood count.

	before TPE	after TPE	Δ before and after TPE (%)	p-value	laboratory range (m/f)
Fibrinogen (mg/dL)	482±58	223±38	-54	0.002	180–350
Antithrombin activity (%)	103±11	54 ± 11	-48	0.0059	70–130
aPTT (s)	28±3	45±8	+60	0.0059	24–36
PT (%)	108±11	68±11	-37	0.002	80–120
INR	0.95±0.06	1.25±0.16	+32	0.0039	0.85–1.15
TT (s)	18±2	20±3	+11	<0.014	14–21
**INTEM**					
CFT (s)	68±26	97±50	+43	0.0144	30–110
MCF (mm)	66±8	58±11	-12	0.0057	50–72
CT (s)	168 (127–292)	172 (135–299)	+2	0.106	100–240
**EXTEM**					
CFT (s)	79±28	134±55	+70	0.002	34–159
MCF (mm)	67±8	57±10	-15	0.0058	50–72
CT (s)	49 (4–64)	52 (2–74)	+6	0.634	38–79
Erythrocytes (x10^12^/L)	4.1±0.7	4.0±0.7	-2	0.0142	3.92–5.08/4.44–5.61
Hemoglobin (g/dl)	12.3±2.1	11.7±2.0	-5	0.0125	11.9–14.6/13.5–16.9
Hematocrit (%)	36.9±5.5	34.6±5.5	-6	0.0039	36.6–44.0/40.0–49.4
WBC (x10^9^/L)	8.2±3.0	8.7±2.9	+6	0.160	4.49–12.68/3.91–10.9
Platelets (x10^9^/L)	286±102	255±89	-11	0.0039	173-390/166-308

P-values are from the Wilcoxon signed rank test; aPTT, activated prothrombin time; PT, thromboplastin time; INR, international normalized ratio; TT, thrombin time; CFT, clot-formation time; MCF, maximum clot firmness; CT, coagulation time; WBC, white blood count.

### Effect of TPE on coagulation parameters

**Fig 2:**
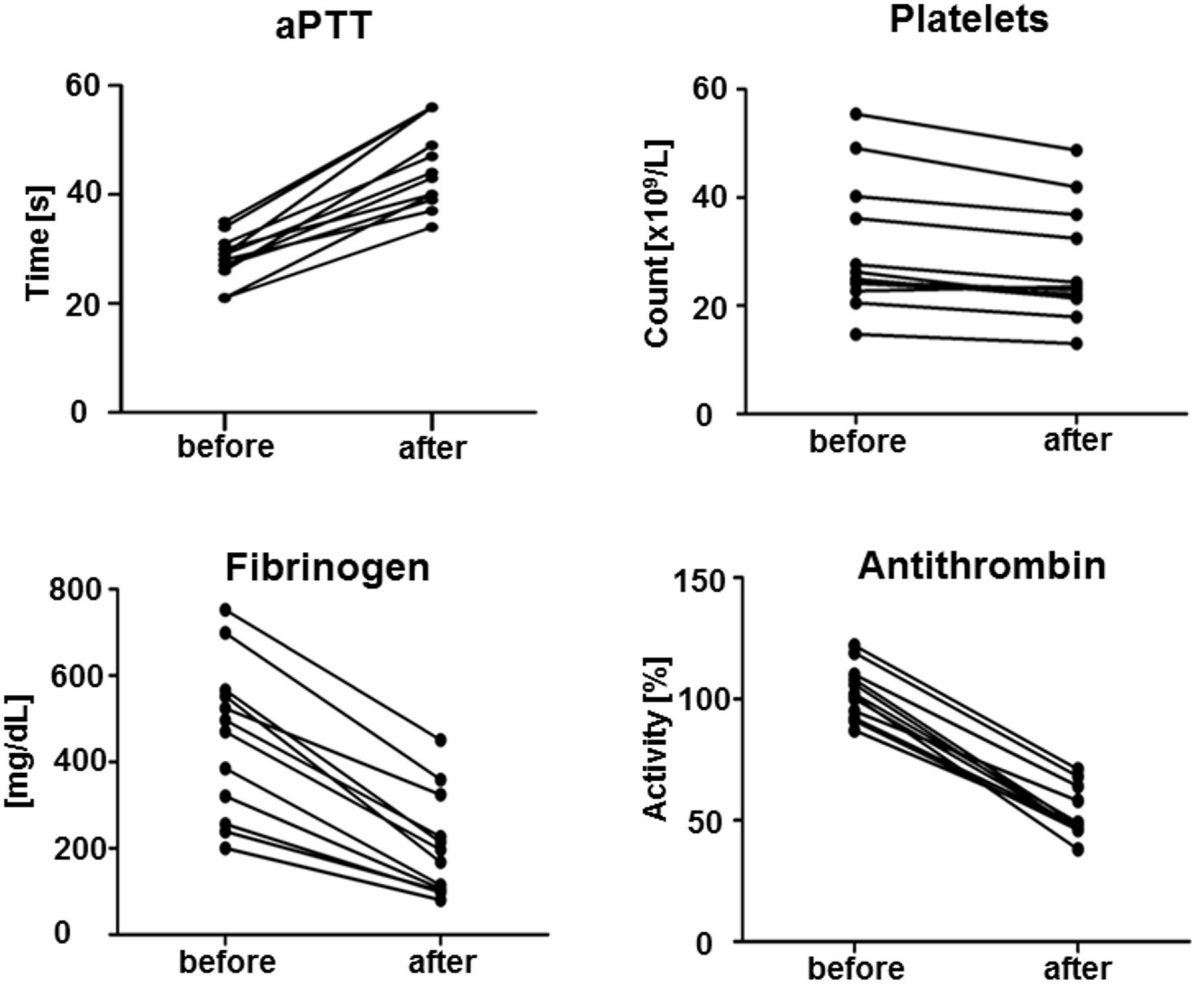
Conventional coagulation factors and platelets before and after therapeutic plasma exchange (TPE) with albumin. After TPE, aPTT (P = 0.0059), fibrinogen (P = 0.002), antithrombin (P = 0.0059) and platelets (P = 0.0039) showed significant changes.

**Fig 3:**
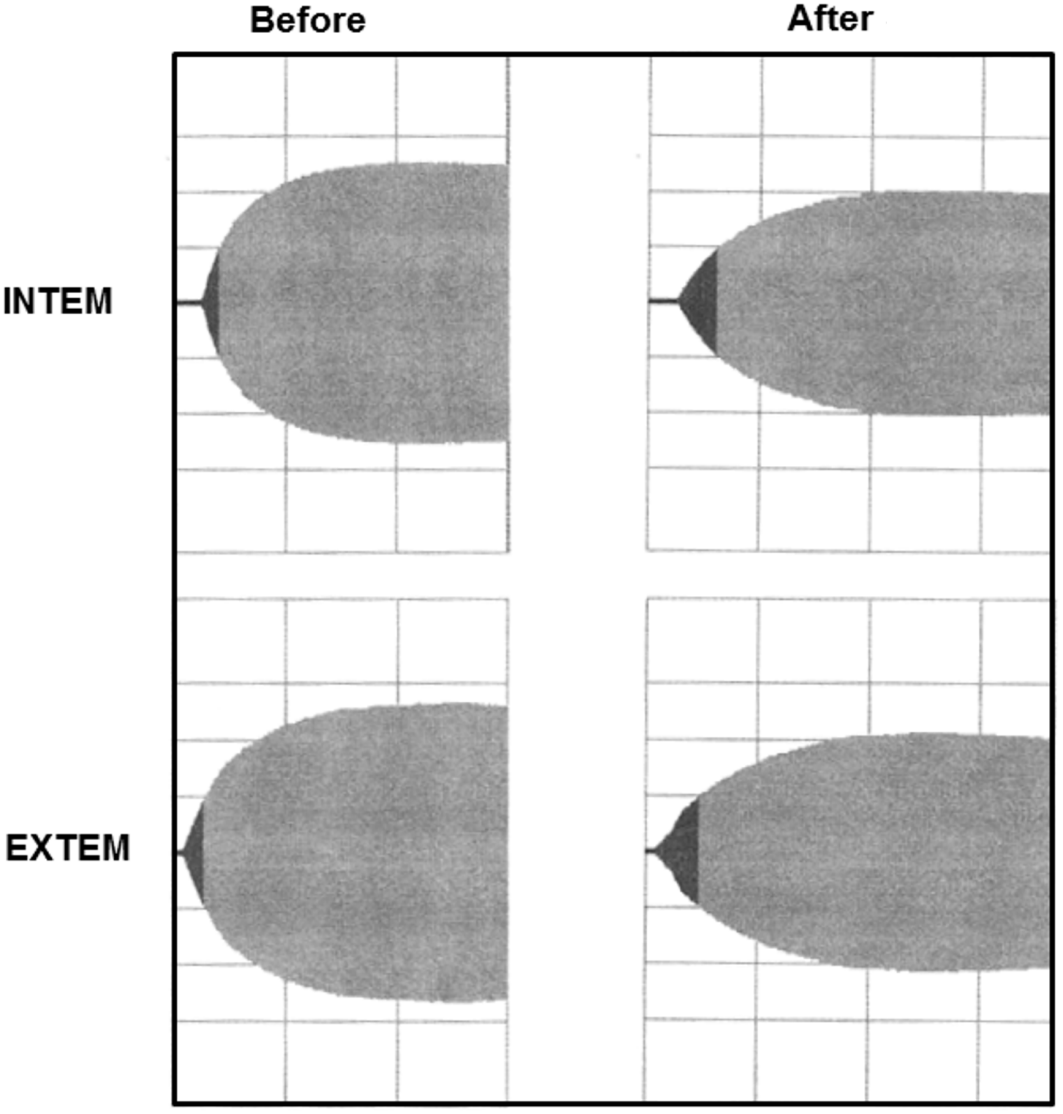
INTEM and EXTEM analyses before and after therapeutic plasma exchange (TPE) with albumin. The thrombelastogram appears elongated and in a narrow shape after TPE. These differences correlate to significant changes in clot-formation time (CFT) and maximum clot firmness (MCF).

**Fig 4:**
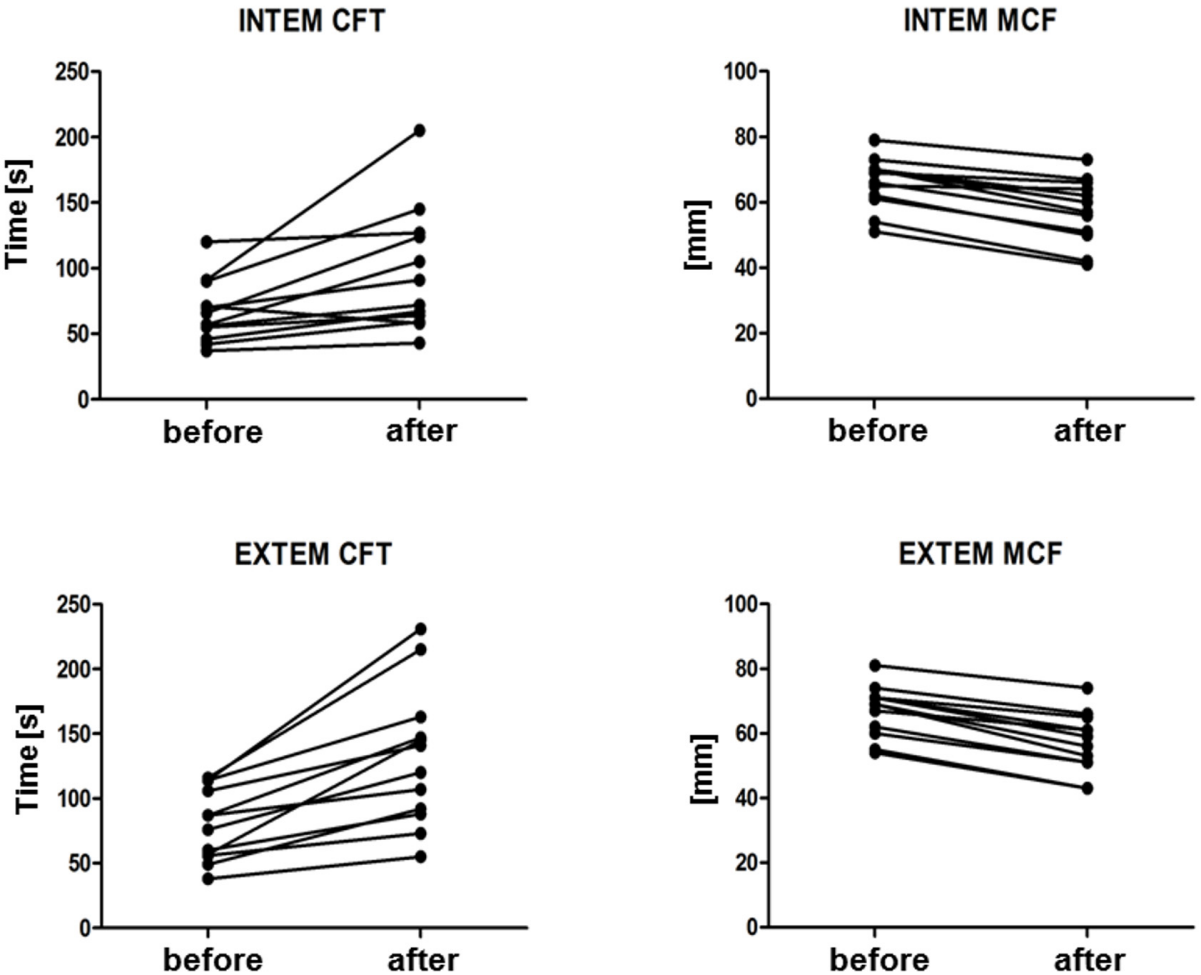
Clot-formation time (CFT) and maximum clot firmness (MCF) of all patients before and after TPE. INTEM and EXTEM analyses revealed a significant prolonged CFT (P = 0.0144 and P = 0.002, respectively) and reduced MCF (P = 0.0057 and P = 0.0058, respectively).

Immediately after TPE, significant decreased values were obtained in erythrocytes (-2%), hemoglobin (-5%), hematocrit (-6%) and platelets (-11%) while white blood count showed an increase (+6%, not significant). Most coagulation parameters changed as hypothesized. Fibrinogen concentration (482 ± 182 vs. 223 ± 122 mg/dL; -54%), antithrombin activity (103 ± 11 vs. 54 ± 11%; -48%), and PT/Quick (108 ± 11 vs. 68 ± 11%; -37%) significantly dropped. Further, aPTT (28 ± 3 vs. 45 ± 8 s; +60%), INR (0.95±0.06 vs. 1.25±0.16; +32%), and TT (18 ± vs. 20 ± 3 s; +11%) changed significantly (Table 2, Fig 2). INTEM analysis revealed significantly prolonged CFT (68 ± 26 vs. 97 ± 50 s; +43%) and reduced maximum MCF (66 ± 8 vs. 58 ± 11 mm; -12%). Similarly, significantly prolonged CFT (79 ± 28 vs. 134 ± 55 s; +70%) and reduced MCF (67 ± 8 vs. 57 ± 10 mm; -15%) were obtained in EXTEM analysis. Neither INTEM nor EXTEM revealed differences in CT (168 (127–292) vs. 172 (135–299) s (+2%) and 49 (4–64) vs. 52 (2–74) s (+6%), respectively (Table 2, Figs 3 and 4).

To sum up, all conventional coagulation parameters changed and showed limited blood coagulation. ROTEM (INTEM and EXTEM) revealed that initiation of clotting remained unaffected while clot formation and firmness are impaired.

## Discussion

Although time-consuming and not evidence-based, fresh frozen plasma is generally preferred over albumin as replacement fluid for TPE immediately before kidney transplantation. Hemorheologic changes after TPE do not differ between both replacement strategies, but in contrast to albumin, plasma replacement avoids depletion of coagulation factors and should therefore minimize the risk of perioperative bleeding [16, 17]. We herein aimed to provide evidence in this regard and estimated the bleeding risk after a TPE using standard coagulation parameters as well as ROTEM POC analysis.

Except an increased fibrinogen concentration, all assessed coagulation parameters were within the normal range in our study population. Fibrinogen increase was mostly due to acute phase response in neuronal inflammation [18]. The fibrinogen level is also elevated in patients suffering from renal insufficiency [19]. Interestingly, this did not translate into a procoagulatory state, but in contrast increased the patients`bleeding risk by inhibition of platelet activation [20]. However, after TPE, fibrinogen concentration dropped. This is well known and some authors propose to monitor fibrinogen and recommend a switch to plasma when levels fall below 1.25 g/L [21–25]. In our study TPE led to a 54% reduction of fibrinogen concentration but in the end fibrinogen remained in the normal range. In addition, we observed significant changes in many others coagulation parameters. Conventional coagulation test showed that PT/Quick dropped. In congruence INR increased and aPTT as well as TT prolonged. However, in our setting using 0.4–1.0 plasma volumes albumin for replacement per session assessed effects are moderate. In cases of higher volume exchanges, aPTT and PT change more dramatically [21]. Thus, TPE with albumin resulted in a significant loss of coagulation factors. Loss can be attributed to removal and to a small proportion to activation by the TPE process. We used centrifugal cell separation in order to minimize possible coagulation activation by hollow fiber plasma filters [26]. Moreover, in the preoperative setting, one would prefer regional anticoagulation with citrate (usually used for centrifugal TPE) over systemic heparin application as used for hollow fiber filtration [22]. Recovery of coagulation parameters takes time e.g. 24h for aPTT and PT and 48–72h for fibrinogen [27]. This is probably of high importance as per se the risk of major bleeding in renal patients is increased [28]. Notably, overall the postoperative hemorrhage rate after kidney transplantation is relevant [29, 30]. Platelets also had been significantly reduced by TPE. Unfortunately, we do not know by simple platelet count analysis if the contact with the TPE equipment affects their function which has been shown previously nor if there are differences in regards to platelet function when comparing TPE with albumin to TPE with fresh frozen plasma [31, 32]. As if matters are not already complicated enough, anticoagulatory factors are also depleted by TPE. We observed a significant, 50% reduction of antithrombin activity after TPE. As antithrombin serves as an indicator substance, one can assume that other factors like protein S or C are depleted as well [23, 24, 33]. Although rare, renal venous thrombosis necessitating transplant nephrectomy is a serious complication [34]. Thus, unfractioned heparin is commonly administered for the perioperative management of renal transplant patients. In this context, reduction of antithrombin might be relevant especially when aPTT is used to adjust the antithrombotic therapy [30, 35].

However, bleeding risk prediction in a kidney recipient is difficult and studies linking single coagulation parameters and perioperative bleeding in kidney transplantation have not been performed. As we are confronted with the disturbance of multiple coagulation parameters after TPE, one should consider performing tests which include a functional analysis of the whole clotting process, especially, when the additional loss of anticoagulatory factors complicates the situation.

ROTEM analysis can assess the entire clotting process, from initiation and clot formation to clot stability, at the bedside and therefore allows timely assessment of bleeding risk and management of hemostatic therapy [15, 36]. ROTEM test is influenced by interactions of platelets and red cells, thereby providing additional information to simple measurements of single factors on patient`s coagulation status. This should probably lead to a more precise estimation of patient`s bleeding risk. As it can be rapidly performed, it might be useful in order to keep graft ischemia time short when one needs to wait for time consuming standard coagulation test after TPE to perform RTx. Therefore, we had chosen to compare standard coagulation parameters to ROTEM for assessment of clotting before and after TPE with albumin. ROTEM has already been shown to effectively detect thrombocytopenia and hypofibrinogenemia in hypocoagulable patients undergoing orthotopic liver transplantation [37–39]. However, if the performance of ROTEM can efficiently reduce blood loss or transfusion need during liver transplantation has to be questioned [37].

In our study, analyses had been performed with extrinsically activated EXTEM assay and intrinsically activated INTEM test [36]. Both tests revealed significantly prolonged CFT after TPE with albumin. MCF, which usually shows an inverse correlation to CFT, was significantly reduced [36]. Notably, CT did not change significantly and is therefore not sufficiently sensitive to estimate bleeding risk after TPE with albumin. Thus, TPE did not significantly alter the initiation of clotting but leads to a distinct reduction of clot formation (CFT) and strength (MCF). This is related to fibrinogen reduction by TPE.

In our study, 2 L albumin 5% (0.4 to 1.0 plasma volumes) were used per TPE session. This procedure displayed the standard method of our center until 2013. Compared to the literature, the replaced albumin volume in our study is within the lower range. Therefore, we have changed our standard protocol in late 2013 according to the guidelines on the use of therapeutic apheresis in clinical practice [40].

Due to the standard protocol of our center, central lines were placed in all patients before TPE. Nevertheless, several studies describe increased complications rates with central lines in this setting [41, 42]. As a less invasive alternative, successful TPE with peripheral venous access was demonstrated even in neurology patients [42, 43]. Central lines offer a continuous high blood flow rate and a straight workflow in the clinical setting. From our experience, the use of central venous catheters is a safe method in the hand of practiced physicians in our clinical setting. In patients before RTx existing arteriovenous fistulae can be used.

### Limitations

This study has limitations. First, it is a small single-center study of patients with neurologic disease which do not reflect the complete complexity of coagulation disturbances of renal patients. Second, the ROTEM-based coagulation testing has not been evaluated for RTx yet. Third, we did not assess clinically meaningful endpoints like postoperative bleeding episodes as we selected a patient population which, for safety reasons, did not undergo surgery after TPE. As we cannot recommend to perform TPE with albumin prior to RTx in regards to our study data, one should evaluate ROTEM after TPE using plasma prior to RTx in further studies [44].

### Conclusion

In summary, we conclude from our data that even TPE with albumin without addition of plasma is able to cause significant disturbances of blood coagulation due to loss of pro- as well as anticoagulant factors. As clot formation and firmness are altered, the bleeding risk following RTx would increase. Thus, in patients needing perioperative TPE, e.g., patients who are immunized or suffer from acute humoral rejection, 100% plasma is recommended as exchange medium for TPE. Moreover, ROTEM is a rapid and easy to perform POC system that detects the induced coagulopathy; however CT measurement is not sufficiently sensitive.
